# Two new species of *Megacanthaspis* Takagi (Hemiptera, Sternorrhyncha, Coccoidea, Diaspididae) from China

**DOI:** 10.3897/zookeys.210.3071

**Published:** 2012-07-24

**Authors:** Jiu-Feng Wei, Ji-Nian Feng

**Affiliations:** 1 College of Life Sciences, Northwest A & F University; 2 Key Laboratory of Plant Protection Resources and Pest Management, Ministry of Education, Entomological Museum, Northwest A & F University, Yangling, Shaanxi Province, 712100, China

**Keywords:** Taxonomy, Sternorrhyncha, Hemiptera, armored scale, China

## Abstract

Two new species of armored scale, *Megacanthaspis hangzhouensis* Wei & Feng, **sp. n.** and *Megacanthaspis hainanensis* Wei & Feng, **sp. n.** are described and illustrated from specimens collected from China. A key to adult female of *Megacanthaspis* species is provided.

## Introduction

Scale insects or superfamily Coccoidea are a diverse group of mostly sap-sucking insects, with at least 30 families and around 8000 species (Andersen 2010). The most species rich family of scale insects is Diaspididae, with over 2400 described species (Ben-dov 2012). Armoured scale insects (Diaspididae) are mainly diagnosed by the extreme modification of the adult females, including the complete loss of the legs, the reduction of the antennae to a single segment (Andersen 2010) and the modification of the abdomen into a specialised pygidium for forming the test. The higher classification within the family is uncertain but two of the major subfamilies are the Aspidiotinae and the Diaspidinae.

The genus*Megacanthaspis* is a small group of scale insects and assigned to the subfamily Diaspidinae, mainly feeding on family Lauraeeae. As presently known, all species were distributed in the Oriental Region and Palearctic region. The localities of *Megacanthaspis* are mapped on Figure 13.

The genus *Megacanthaspis* was originally established by Takagi (1961) to accommodate a species from Japan. Takagi (1961) characterized the genus as follows: ‘It is particularly characterized by having very prominent, conical, glanduliferous processes along the margin of the abdomen.’ These features separate the genus *Megacanthaspis* from other genera such as *Mercetaspis* Gomez-Menor, 1927 (Takagi 1961).

Takagi (1970) reported the species *Megacanthaspis litseae* collected from Taiwan of China and later he (1981) revised the genus, added two new species from Japan and transferred the species *Nanmuaspis phoebia* Tang, 1977, collected in China into *Megacanthaspis*.

Recently, two further species of *Megacanthaspis* were foundand are described and illustrated herein, bringing the total number of species in the genus to 7 species. A key to all known species of *Megacanthaspis* is provided. Moreover, a new host belongs to Poaceae is record.

## Materials and methods

Slide-mounted specimens, mounted in Canada balsam using the method discussed by Henderson (2011), were studied.

The morphological terminology used in the descriptions mainly follows that of Takagi (1981) which also has illustrations of most of other species included in the genus. The illustrations were drawn from slide-mounted adult females specimens and depicted with the dorsum on the left and venter on the right. All measurements were given in micrometer (μm) and were made using NIT-Elements D. All specimens are deposited in the Entomological Museum, Northwest A & F University, Yangling, Shaanxi, China (NWAFU).

### Checklist of known species of the genus *Megacanthaspis* Takagi

*Megacanthaspisactinodaphnes* Takagi, 1961; Japan.

*Megacanthaspis hangzhouensis* sp. n.; China (Hangzhou).

*Megacanthaspis hainanensis* sp. n.; China (Hainan).

*Megacanthaspis langtangana* Takagi, 1981; Nepal.

*Megacanthaspis leucaspis* Takagi, 1981; Japan.

*Megacanthaspis litseae* Takagi, 1970; China (Taiwan).

*Megacanthaspis phoebia* (Tang, 1977); China (Zhejiang).

## Taxonomy

### Genus Megacanthaspis

Takagi, 1961

http://species-id.net/wiki/Megacanthaspis

Megacanthaspis Takagi, 1961: 97. Type species: *Megacanthaspis actinodaphnes* Takagi, original designation.

#### Generic diagnosis.

**Female scale.** Brown to dark brown, elongate, high convex; exuvia apical. **Male scale.** white, approximately parallel sides, slightly convex.

**Adult female.** Body outline elongate, derm membranous. **Cephalothorax.** Antennae each with a long seta and a tubercle. Anterior spiracles each with a group of trilocular pores, some species also with pores near posterior spiracles. **Pygidium.** Pygidium rounded along posterior margin, with a series of serrate processes or plates, none of which are sclerotized enough to call lobes.In certain species, this processes or plates degenerate or invisible. **Marginal gland spines** occurring on the abdomen, each associated with 1 or more microducts. **Gland tubercles** present or absent,if present, near both anterior and posterior spiracles, others occurring submarginally of abdominal segments I–III. **Ducts.** Dorsal macroducts short, 2- barred, with the orifice surrounded by a sclerotized rim, forming obscure segmental rows in some species. Ventral microducts as large as or smaller than dorsal ducts. **Anal opening** situated on centre of pygidium. **Perivulvar pores** quinquelocular, present in an arc, sometimes divided into a median group and two lateral groups.

#### Distribution.

Palaearctic and Oriental regions.

#### Key to adult female *Megacanthaspis* Takagi

**Table:** 

1	Marginal gland spines present on segment II	2
–	Marginal gland spines absent on segment II	3
2	The posteriormost pair appressed together at apex of pygidium	*Megacanthaspis litseae* (Takagi)
–	The posteriormost pair widely separated from each other	*Megacanthaspis langtangana* (Takagi)
3	Marginal gland spines present on segment III	4
–	Marginal gland spines absent on segment III	5
4	The posteriormost pair appressed together at apex of pygidium	*Megacanthaspis actinodaphnes* (Takagi)
–	The posteriormost pair widely separated from each other	*Megacanthaspis phoebia* (Tang)
5	Marginal gland spines absent on segment IV	*Megacanthaspis hangzhouensis* sp. n.
–	Marginal gland spines present on segment IV	6
6	Marginal gland spines each associated with 1 microduct	*Megacanthaspis leucaspis* (Takagi)
–	Marginal gland spines each associated with 2-4 microducts	*Megacanthaspis hainanensi*ssp. n.


### Megacanthaspis hangzhouensis

Wei & Feng sp. n.

urn:lsid:zoobank.org:act:C2F9DCB3-51BB-494E-B298-5ABE9A9001A5

http://species-id.net/wiki/Megacanthaspis_hangzhouensis

Figures 1–6

#### Material examined.

**Holotype**: adult female: CHINA, Zhejiang Prov., Hangzhou City, Hangzhou botanical garden, 30°25'N, 120°12'E, 1.5.1982, Chou (NWAFU).

Paratypes: 7 adult females: same data as the holotype (NWAFU).

#### Description, n=8.

**Adult female.** Appearance in life not recorded. Slide-mounted adult female 552–617 μm long (holotype 598 μm long); 309-362 μm wide (holotype 337 μm wide), body outline oblong oval, with indistinct segmentation. **Cephalothorax.** Antennae each with a long seta and a tubercle. Anterior spiracles with 1-2 trilocular pores, pores absent from posterior spiracles. **Pygidium** marginal processes degenerate. Pygidial lobes absent, without paraphyses and plates. **Marginal gland spines** each 14-19 μm long, in 6 pairs on abdominal V-VIII, 1 pair on abdominal segments VII and VIII and 2 pairs on abdominal segments V and VI, each associated with 1 microduct; posteriormost median pair of gland spines widely separated. **Gland tubercles** absent. Dorsal macroducts forming obscure segmental rows and not obviously divided into marginal, submarginal and submedial groups, with about 17 on each side; without marginal dorsal macroducts at apex of pygidium between the posteriormost gland spines. **Ventral microduct** is smaller than dorsal macroduct, few, scattered on cephalothorqx and abdomen, with 4 or 5 near each anterior and posterior spiracles. **Anal opening** separated from apex of pygidium by a space about 82 μm long. **Perivulvar** **pores** present in an arc, divided in 5 groups, 4–7 median group, 5–8 anterolaterally, and 7–10 posterolaterally, 28–43 in total.

#### Diagnosis.

The new species is very close to *Megacanthaspis phoebia* (Tang, 1977) in having 6 pairs marginal gland spines. But differs in having (character-states on *Megacanthaspis phoebia* in brackets): (i) 2 pairs of gland spines on abdominal segments V and VI (only single on segments V & VI); (ii) marginal dorsal macroducts absent from apex of pygidium between median gland spines (present); (iii) gland tubercles absent (present).

#### Host.

*Pleioblastus amarus* (Poaceae).

#### Etymology.

Named after Hangzhou, the type locality.

#### Distribution.

China (Zhejiang).

**Figures 1–6.**
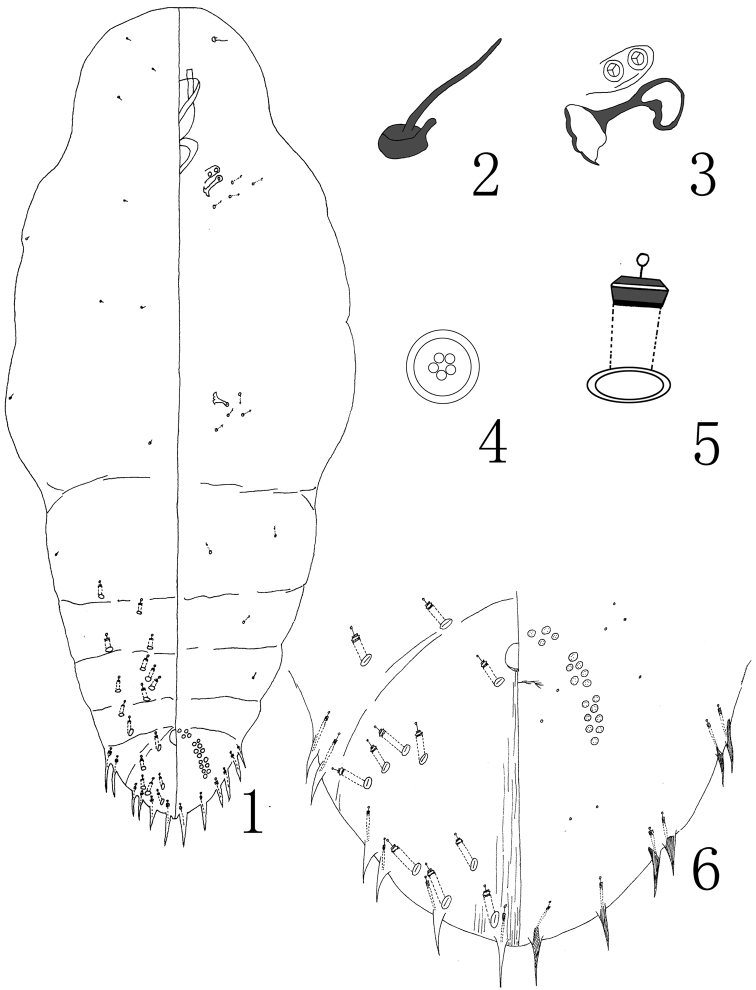
Megacanthaspis hangzhouensis Wei & Feng, sp. n., adult female: **1** habitus **2** detail of antenna **3** detail of anterior spiracle **4** quinquelocular pores **5** dorsal 2-barred duct **6** pygidium.

### Megacanthaspis hainanensis

Wei & Feng sp. n.

urn:lsid:zoobank.org:act:1B66EA08-618D-47FD-B808-0A3BB90D6901

http://species-id.net/wiki/Megacanthaspis_hainanensis

Figures 7–12

#### Material examined.

**Holotype**: adult female: CHINA, Hainan Prov., Gaotuo mountain, 19.5.1963, Chou (NWAFU).

Paratypes: 12 adult females, same data as the holotype (NWAFU).

#### Description.

**N=13. Adult female.** Appearance in life was not recorded. Slide-mounted adult female 513–597 μm long (holotype 577 μm long); 199–209 μm wide (holotype 209 μm wide), body outline fusiform, with obscure segmentation. **Cephalothorax.** Antennae each with a long seta and a tubercle. Anterior spiracles each with 2–4 trilocular pores; pores absent from posterior spiracles. **Pygidium** with serrate process (plates) on abdominal segments VI-VIII, lobes absent, plates arranged 2, 3, 3 among the marginal gland spines, without paraphyses. **Marginal gland spines** each 9.93–18.9 μm long, in 5 pairs on abdominal IV-VIII, more or less enlarged, each associated with 2–4 microducts, median pair widely separated. **Gland tubercles** present on prothrax, metathrax and abdominal segment I-II, each with 1 microducts. **Dorsal macroducts** present on abdominal segments I-VIII; forming more or less segmental rows on abdominal segments I-VI, but scattered on abdominal segments VII-VIII; with a macroduct between median gland spines. **Ventral macroducts** 2-barred, as big as dorsal macroducts, scattered occurring on lateral body margin on prothorax, metathorax and abdominal segments I-IV. Ventral microducts present on prothorax, metathorax, segments I-IV. **Anal opening** about 68 μm long from apex. **Perivulvar pores** in an arc with a total of 13–25.

#### Diagnosis.

The new species is very similar to *Megacanthaspis phoebia*, but can be distinguished by by having (character-states on *Megacanthaspis phoebia* in brackets): (i) 5 pairs of marginal gland spines (6 pairs); (ii) a macroduct present medially between the median gland spines (absent).

#### Etymology.

Named after Hainan, the type locality.

#### Distribution.

China (Hainan).

**Figures 7–12.**
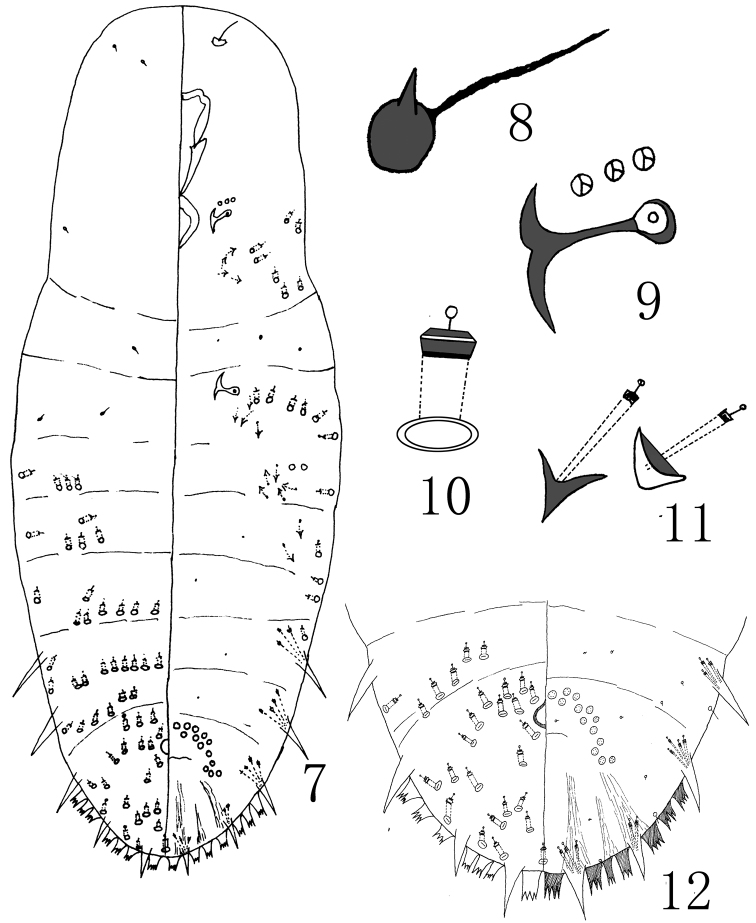
Megacanthaspis hainanensis Wei & Feng, sp. n., adult female: **7** habitus **8** detail of antenna **9** detail of anterior spiracle **10** dorsal 2-barred duct **11** detail of 2 gland tubercles **12** pygidium.

**Figure 13.**
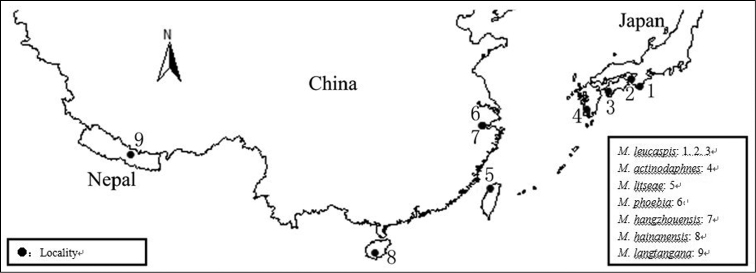
Distribution of Megacanthaspis.

## Supplementary Material

XML Treatment for Megacanthaspis

XML Treatment for Megacanthaspis hangzhouensis

XML Treatment for Megacanthaspis hainanensis
